# Network discovery with large DCMs

**DOI:** 10.1016/j.neuroimage.2012.12.005

**Published:** 2013-03

**Authors:** Mohamed L. Seghier, Karl J. Friston

**Affiliations:** The Wellcome Trust Centre for Neuroimaging, UCL, 12 Queen Square, London, WC1N 3BG, UK

**Keywords:** Effective connectivity, Functional MRI, Dynamic causal modelling, Connectivity, Graph theory, Bayesian

## Abstract

In this work, we address the problem of using dynamic causal modelling (DCM) to estimate the coupling parameters (effective connectivity) of large models with many regions. This is a potentially important problem because meaningful graph theoretic analyses of effective connectivity rest upon the statistics of the connections (edges). This calls for characterisations of networks with an appreciable number of regions (nodes). The problem here is that the number of coupling parameters grows quadratically with the number of nodes—leading to severe conditional dependencies among their estimates and a computational load that quickly becomes unsustainable. Here, we describe a simple solution, in which we use functional connectivity to provide prior constraints that bound the effective number of free parameters. In brief, we assume that priors over connections between individual nodes can be replaced by priors over connections between modes (patterns over nodes). By using a small number of modes, we can reduce the dimensionality of the problem in an informed way. The modes we use are the principal components or eigenvectors of the functional connectivity matrix. However, this approach begs the question of how many modes to use. This question can be addressed using Bayesian model comparison to optimise the number of modes. We imagine that this form of prior – over the extrinsic (endogenous) connections in large DCMs – may be useful for people interested in applying graph theory to distributed networks in the brain or to characterise connectivity beyond the subgraphs normally examined in DCM.

## Highlights

► Here we investigate the possibility to invert large DCMs with many regions. ► To do that, we place constraints on priors to bind the number of free parameters. ► Constraints are provided by the principal modes of the functional connectivity. ► Bayesian model comparison is used to identify the optimal number of modes. ► The ability to invert large DCMs provides a new opportunity for graph theory users.

## Introduction

Usually, analyses of directed (effective) connectivity using dynamic causal modelling (DCM) (Friston et al., 2003) on fMRI data consider a small number of regions (e.g., less than 10 regions), typically excited by carefully designed experimental manipulations (for a review see (Friston, 2011b; Seghier et al., 2010b)). In terms of graph theory, this corresponds to a characterisation of subgraphs that are exposed to exogenous input—where these inputs can be experimental (stimulus) functions or random fluctuations (Valdes-Sosa et al., 2011). Recently, there has been growing interest in the modelling of larger networks or graphs, which we take to imply graphs with sixteen or more regions (Bullmore and Sporns, 2009; Guye et al., 2010; He and Evans, 2010). These analyses are usually required to contextualise the activity in subgraphs, within the setting of a larger distributed network—or to provide estimates of effective connectivity for subsequent characterisation with graph theory measures such as characteristic path length, modularity, centrality and network resilience (Rubinov and Sporns, 2010).

However, increasing the number of regions or nodes in a DCM presents some problems. Clearly, the number of extrinsic (between-node) connections or edges increases with the square of the number of nodes. This can lead to models with enormous numbers of free parameters and profound conditional dependencies among the parameters (Daunizeau et al., 2011). Furthermore, the computational time required to invert these models grows exponentially with the number of free parameters. In this paper, we present a simple solution to the inversion of large DCMs with sixteen or more regions. This solution is based on plausible priors that effectively constrain the number of extrinsic coupling parameters. Crucially, the plausibility of these priors can be established by Bayesian model comparison (Penny et al., 2004, 2010).

This approach is based upon the hypothesis that distributed brain responses are mediated by coupling among spatial patterns or modes (Friston, 2009), of the sort seen in resting state functional connectivity studies; see for example (Biswal et al., 2010; Damoiseaux et al., 2006; Smith et al., 2009; Yeo et al., 2011). This means that the connections among individual regions can be replaced by connections among modes. Because we can control the number of modes, one can place an upper bound on the number of extrinsic coupling parameters that need to be estimated. Crucially, this hypothesis can be tested by examining the evidence for models with different numbers of modes, where – in the limiting case that the number of modes and nodes are the same – we return to the conventional (unconstrained) priors. This constraint pertains to, and only to, extrinsic connections: each node or region can still have its own intrinsic connectivity and parameters of its (regionally specific) hemodynamic response function. This means, we do not simply model the coupling among modes but rather use the modes to place prior constraints on, and only on, extrinsic connections. In what follows, we describe this approach in detail for deterministic DCM and evaluate the underlying hypothesis using Bayesian model comparison. In brief, we test the notion that distributed brain responses can be explained by extrinsic coupling among modes by comparing the Bayesian model evidence for models whose priors entertain an increasing number of modes. If the hypothesis is correct, then we would expect to see model evidence peak at a particular number of modes and then decline again as the model becomes over parameterised.

We envisage that the resulting search over models (number of *a priori* modes) could be applied to any fMRI time series using DCMs with full extrinsic connectivity. The model with the greatest evidence can then be pruned using post-hoc model optimisation (Friston et al., 2011) to provide posterior estimates for subsequent characterisation of the resulting directed graphs. We will illustrate these procedures using empirical data.

This paper comprises three sections. In the first, we provide a brief overview of dynamic causal modelling for functional magnetic resonance imaging (fMRI), with a special focus on the priors over extrinsic connectivity parameters—and how they are constructed using the eigenvectors (principle components or modes) of the functional connectivity matrix. In the second section, we describe the fMRI data used to illustrate the approach and the DCM used to model these data. In the final section, we perform a Bayesian model comparison over models with different priors to establish the optimal number of modes. We conclude with a discussion of the behaviour of model evidence and the structure of the reduced model after post-hoc optimisation.

## Methods

### Dynamic causal modelling

In this section, we provide a brief review of dynamic causal modelling for fMRI, paying special attention to the prior constraints on the extrinsic connectivity parameters. These Gaussian priors are based upon the principal components or eigenvectors of the functional connectivity matrix—or sample correlation matrix of the time-series data. We will see that the ensuing priors can be formulated in a reasonably straightforward fashion using Kronecker tensor forms.

DCM for fMRI rests on a generative model that has two components: (i) a neuronal model describing interactions (dependencies) in a distributed network of neuronal populations, and (ii) a forward biophysical model that maps neuronal activity to observed hemodynamic responses. The default implementation in deterministic DCM for fMRI models the rate change in neuronal activity according to the following bilinear evolution or state equation (Friston et al., 2003):

(1) $$\frac{dz}{dt}\;=\;\left(A\;+\;\sum_{i=1}^{k}u_{i}B^{\left(i\right)}\right)z\;+\;Cu$$

where *z*(*t*) is the (lumped) activity of the neuronal populations in a given node, *A* is the first-order (endogenous or average) connectivity in the absence of inputs, *B* is the second-order interaction between activity and input (bilinear or modulatory effects), and *C* mediates the effects of (exogenous or experimental) inputs *u*(*t*) on activity. The set of neuronal connectivity parameters (*A*,*B*,*C*) and the hemodynamic parameters *H* of the forward model are noted as vector *θ*.

The posterior probabilities of the model parameters *θ* = (*A*,*B*,*C*,*H*) are assessed with Bayesian inversion using standard variational techniques based upon the Laplace approximation (Friston, 2002; Friston et al., 2002, 2007a). In this Bayesian inversion framework, two quantities are estimated (e.g. (Daunizeau et al., 2011)): (i) the posterior distribution over model parameters *p*(*θ*|*M*,*Y*), which can be used to make inferences about model parameters *θ* of model *M* given data *Y*, and (ii) the probability of the data given the model *p*(*Y*|*M*), known as the model evidence or marginal likelihood. The log evidence is approximated by a negative (variational) free energy that is used for Bayesian model comparison or scoring (e.g. (Penny, 2012)). Further details about DCM for fMRI responses can be found elsewhere (Friston, 2011b; Seghier et al., 2010b).

#### Priors on the parameters of DCM for fMRI

In this work, we used the latest release of DCM (noted DCM12) on MATLAB 2010a (MathWorks, Natick, Massachusetts USA). This release incorporates priors on the precision of observation noise with an expected log-precision of four. This simply encodes the prior belief that there is a reasonable signal to noise ratio in the fMRI timeseries, which generally leads to more robust model inversion, with typical fMRI responses.

Gaussian priors on the connectivity and hemodynamic parameters are referred to as “shrinkage” priors (Friston et al., 2003) because they tend to “shrink” posterior means to their prior expectation. They are specified in terms of a prior mean and covariance. The prior means and covariances on the endogenous (extrinsic and intrinsic) connectivity parameters usually depend on the number of nodes (*n*) in the model. In this context, the prior precision increases with the number of nodes to preclude runaway excitation—which makes the shrinkage priors particularly stringent for larger DCMs. More specifically, the priors on self-connections and the between-node connections in DCM12 are:

(2) $$p\left(A_{\mathrm{ij}}|M\right)\;=\;N\left(\eta_{\mathrm{ij}},\upsilon_{\mathrm{ij}}\right)\;=\;\{\begin{array}{c} N\left(-1/2,1/\left(8\cdot n\right)\right)\;i=j\\ N\left(1/\left(64\cdot n\right),8/n\right)\;i\neq j \end{array}$$

This leads to a diagonal form for the prior covariance $\Sigma \subset R^{n\cdot n\times n\cdot n}$ over all the average connectivity parameters:

(3) $$\begin{array}{c} p\left(\text{vec}\left(A\right)|M\right)\;=\;N(\text{vec}(\eta ),\Sigma )\\ :\Sigma \;=\;\text{diag}(\text{vec}(\upsilon )) \end{array}$$

This diagonal form means that we have no prior beliefs about correlations among the intrinsic and extrinsic connectivity parameters in the $A\subset R^{n\times n}$. However, for large DCMs (e.g., *n* > 8), we can now introduce correlations to decrease the effective number of free parameters, by reducing the rank of the prior covariance matrix. To do this we need to find some plausible priors or constraints. Here these constraints are based upon the principal components or eigenvectors of the functional connectivity matrix. These define the number of modes (*m*) that will constrain the rank of the prior covariance. The prior covariance is constrained as follows:

Let *Y* = [*y*_1_,*y*_2_,*y*_3_,…*y_n_*] be the set of observed BOLD responses in *n* nodes where *y_i_* is a timeseries from the *i*th node. This is itself usually the principal component over a number of local voxels that constitute the region. Furthermore, *y_i_* is generally adjusted by removing confounds (e.g. signal drift, session effects, etc.). We start using singular value decomposition to find the principal modes:

(4) $$Y\;=\;U\cdot S\cdot V^{T}$$

where the diagonal elements of S contain singular values (i.e. mode amplitudes), $U\subset R^{n\times n}$ contains the modes or singular vectors, and *V* contains the singular variates. We then select the modes with the largest singular values $U_{m}\subset R^{n\times m}$ and use them to remove minor modes from the prior covariance as follows:

(5) $$\begin{array}{c} \Sigma_{m}\;=\;K_{m}\cdot \Sigma \cdot K_{m}^{T}\\ K_{m}\;=\;\left[U_{m}\cdot U_{m}^{T}\right]⊗\left[U_{m}\cdot U_{m}^{T}\right] \end{array}$$

where ⊗ denotes the Kronecker tensor product. Effectively, the projector matrices *K*_*m*_ induce prior correlations among the connections and ensure certain mixtures of parameters have no prior variance—so that they are essentially fixed. The *∑_m_* thus reflects the constrained prior covariance over all endogenous connectivity parameters in DCMs with *m* modes.

It is important to keep in mind that these constrained priors pertain only to endogenous connectivity parameters. Prior beliefs about the bilinear, exogenous input and hemodynamic parameters are unchanged and thus each region can still have its own hemodynamic response function. It is also worth noting that priors on the noise precision at each node remain independent (for more details see (Daunizeau et al., 2009; Friston et al., 2007a; Li et al., 2011)). Note that the use of modes – as prior constraints on coupling among nodes – is similar to but formally distinct from modelling the coupling among modes *per se*; e.g. (Stevens et al., 2007)). In other words, the DCM is still trying to explain region-specific activity in each node (as opposed to the activity of modes). This concludes our description of how functionally informed modes of activity are used to place constraints on the effective number of coupling parameters. In the next section, we consider the optimal number of principal modes, in terms of model evidence, using real fMRI data.

### Empirical fMRI data and dynamic causal models

This section describes the empirical data used for model inversion and comparison. We describe the experimental design, the selection of regions or nodes and the ensuing dynamic causal model. The empirical data came from a block-design fMRI activation study of 10 healthy subjects described in (Seghier and Price, 2010; Seghier et al., 2008). During two separate scanning sessions, subjects were asked to (i) read aloud 96 three to six letter object names with consistent spelling-to-sound relationships (e.g. hat, tent, horse, carrot); (ii) name presented pictures of familiar objects; (iii) say “1,2,3” to meaningless pictures of symbols or non-objects (unfamiliar stimuli). Each session comprised four different word reading blocks that lasted 18 s, with 12 words per block presented at a rate of three words every 4.5 s (i.e. as triads), four blocks of object naming presented at the same rate as during reading, four blocks of saying “123” to unfamiliar (meaningless) pictures of symbols or non-objects, and six blocks of fixation (14.4 s per fixation block). To minimise artefacts from head motion, subjects were asked to whisper their response with minimal mouth movement. Stimulus presentation was via a video projector, a front-projection screen and a system of mirrors attached to a head coil. Additional details about the paradigm and stimuli can be found in our previous descriptions of this study (c.f. (Kherif et al., 2009; Seghier and Price, 2010; Seghier et al., 2008)).

#### fMRI data acquisition and analysis

Data were acquired on a 1.5 T scanner (Siemens Medical Systems, Erlangen, Germany). Functional imaging consisted of an EPI GRE sequence (TR/TE/Flip = 3600 ms/50 ms/90°, FOV = 192 mm, matrix = 64 × 64, 40 axial slices, 2 mm thick with 1 mm gap). Data processing and statistical analyses were performed with the Statistical Parametric Mapping SPM software package (Wellcome Trust Centre for Neuroimaging, London UK, http://www.fil.ion.ucl.ac.uk/spm/). All functional volumes were spatially realigned, un-warped, normalized to the MNI space using a unified normalisation–segmentation procedure and smoothed with an isotropic 6-mm FWHM Gaussian kernel, with final voxel sizes of 2 × 2 × 2 mm. The pre-processed functional volumes of each subject were then submitted to a fixed-effects analysis, using the general linear model at each voxel. Each stimulus onset was modelled as an event, encoded in condition-specific “stick-functions” with an inter-stimulus interval of 4.5 s and duration of 4.32 s per trial. Each block contained four successive events. The resulting stimulus functions were convolved with a canonical hemodynamic response function to form regressors for the linear model. Confounding regressors included drift terms and head motion. The appropriate summary or contrast image was then entered into a second-level (between subject) analysis (i.e. random-effects analysis) to enable inferences about regional responses at the group level in the usual way. These were used to constrain the subject specific regions or nodes for subsequent DCM analysis.

#### Selection of nodes

**Fig. 1:**
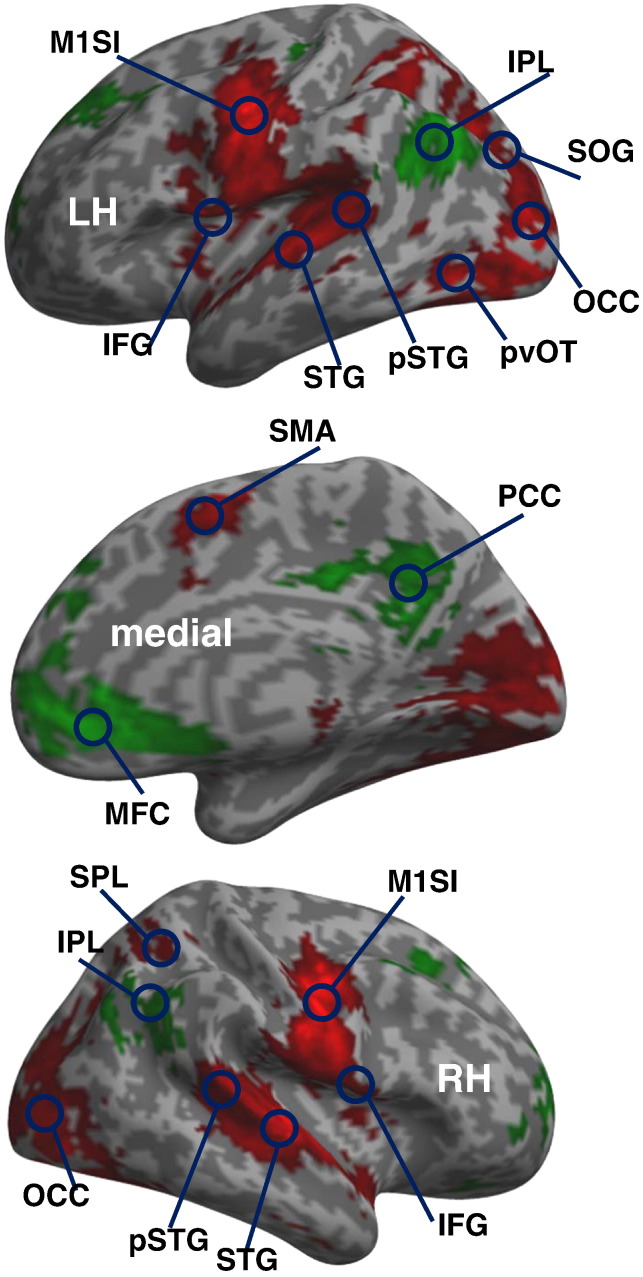
Illustration of the regions of interest for the contrast “reading > fixation” (in red) and “fixation > all tasks” (in green) at *p* < 0.05 FWE-corrected in a group level whole brain SPM analysis. LH = left hemisphere, RH = right hemisphere. See Table 1 for a list of the anatomical location and MNI coordinate of these 20 regions.

Based on the group analysis, we identified a total of 20 regions of interest that showed significant effects across subjects at *p* < 0.05 FWE-corrected over the whole the brain; see Fig. 1 for their locations and Table 1 for a full list of coordinates. These regions were chosen to cover a distributed bilateral network from visual cortex to articulatory motor regions. This region selection ensured that the regional responses were defined functionally, in a sense that they showed significant task-related effects. Here, we used two contrasts of interest: the first was “reading > fixation” that identified 16 regions with strong activations during reading aloud relative to fixation – including a large bilateral set of visual, auditory, phonological and articulatory regions (Fiez and Petersen, 1998; Price, 2000). The second contrast was “fixation > all tasks” and identified four task-induced deactivations that are part of the well-known default mode network (Greicius and Menon, 2004; Raichle et al., 2001)—including bilateral inferior parietal lobule, posterior cingulate cortex and medial prefrontal cortex (Fig. 1). Our rationale – of selecting nodes with both task-induced activations and deactivations – was to ensure positive and negative posterior estimates of the coupling parameters and to explicitly model interactions between functionally heterogeneous networks (e.g. (Andrews-Hanna et al., 2010; Graves et al., 2010; Laird et al., 2009; Richardson et al., 2011; Seghier and Price, 2009; Seghier et al., 2010a; Wirth et al., 2011)).

After defining our 20 regions from the group analysis, principal eigenvariates (i.e., summary time series) were extracted from each subject (using voxels in subject-specific SPMs that survived a criteria of *p* < 0.05 uncorrected) around the subject-specific maximum that was closest to the coordinates of the group maximum in Table 1. Regions were extracted for each session separately within an 8 mm radius sphere and the principal eigenvariates were adjusted for confounds. The resulting summary time series from each region were concatenated over the two sessions for dynamic causal modelling.

#### Specification of the DCM

The dynamic causal model for each subject's (concatenated) time series specified as follows: (i) we assumed that visual information entered at the level of bilateral occipital regions (low-level visual areas, Table 1). Thus the driving input (i.e. words, objects and unfamiliar stimuli) was connected to the two occipital regions, (ii) all between-node (extrinsic) connections were considered, resulting in a fully-connected model. In this illustrative analysis we ignored condition and stimulus specific effects—treating the activations as due to simple stimulus processing. This meant that we did not specify any modulatory or bilinear parameters. In summary, our fully connected DCM comprised 20 nodes, where the response of each node was summarised by its principal eigenvariate of 198 observations. Note that this model was not designed to test hypotheses or cognitive models of word reading—it is a simple model that was sufficient to focus on the prior constraints specified in terms of the number of principle modes.

#### Prior constraints

**Fig. 2:**
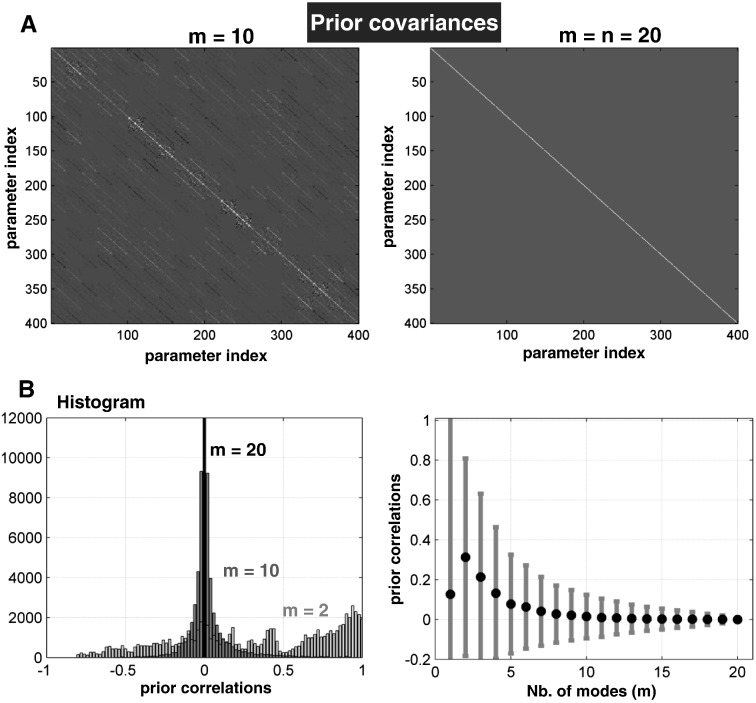
(A) The prior covariances matrix: the 400 endogenous connectivity parameters with constrained priors at *m* = 10 (left) or without constraints (right) of a typical subject. (B) The use of functional connectivity to constrain the priors induced strong prior dependencies among parameters—that increased with the number of minor modes removed. This is illustrated by transforming the covariances to correlations: (left) illustration of histograms of correlations for *m* = 2 and *m* = 10; (right) mean correlations (black dots) and their standard deviations (gray vertical lines) at each number of modes from *m* = 1 to *m* = 20.

As noted above, removing the minor modes from the priors on the connectivity parameters induces prior dependencies among endogenous coupling parameters. Fig. 2A illustrates this in terms of the prior covariance matrix – from one typical subject – after removing the minor modes. The dependencies between the parameters appear as off-diagonal covariances in Fig. 2A, which increases with the number of modes removed (Fig. 2B). These prior constraints mean that the prior covariances are no longer fixed and may differ between subjects.

#### Bayesian model comparison

To test the hypothesis that the fMRI data can be explained in terms of constrained coupling among modes or patterns of nodes, we inverted the DCM above using an increasing number of modes, with *m* varying from 1 to *n*. This resulted in 20 DCMs per subject. In other words, the number of minor modes removed from the priors in our models varied between 0 to *n* − 1. We expected to see the evidence for each model increased initially, with the number of modes and then decreased again as redundant coupling parameters rendered the model overly parameterised and thus too complex. Note that if the data were caused by unconstrained coupling among individual nodes, the model with the largest number of modes could have the most evidence. Put another way, the very existence of a peak in the log evidence – as a function of the number of modes – provides evidence in favour of the hypothesis upon which our prior constraint rests.

All 20 dynamic causal models of the data from our 10 subjects were inverted using variational Bayesian procedures as described above (Friston et al., 2007a, 2007b). This provided posterior parameter estimates and a free energy bound on the log evidence for each model. The log evidence can be regarded as the difference between accuracy and complexity (c.f. Equations [19–23] in (Penny, 2012), where accuracy is simply the log likelihood of the data expected under the posterior estimates. Roughly speaking, the complexity can be regarded as the number of free parameters that are required to explain the data accurately. Our prior constraints therefore place an upper bound on model complexity and, in principle, could provide models for which there is greater evidence.

#### Post-hoc model optimisation

Post-hoc model optimisation or network discovery (Friston et al., 2011) is a recent Bayesian procedure that can infer the functional architecture of distributed systems using DCM after inverting one fully connected model. It uses Bayesian model selection to assess the impact of absent edges or connections (i.e. discover the sparsity structure) in a graph that best explains the observed time-series. Its core algorithm is based on a proxy (post-hoc) scheme for scoring a huge number of models, based on the posterior density of a single fully connected model. This scoring can then identify the (reduced) model that has the highest evidence or marginal likelihood (Friston et al., 2011). Here, post-hoc optimisation or reduction was applied to the model with the optimal number of modes. Note that the post-hoc model optimisation operates at the individual subject level and thus the structure of the reduced model at the group level was obtained using Bayesian parameter averaging over our 10 subjects, as implemented in DCM12.

### Synthetic fMRI data

Finally, we used simulations to quantify the behaviour of the posterior estimates of the coupling parameters, when the number of modes is reduced. Clearly, to assess any bias in the parameter estimates due to prior covariance constraints, we needed to know their true value. Therefore, we used the same procedure as in Penny (2012) to generate synthetic fMRI timeseries for 20 nodes, with known coupling parameters. These data were created using the “spm_dcm_generate” routine of DCM12, under the following conditions: (i) the stimulus functions of our fMRI experiment (detailed above) were used as driving inputs. This produced synthetic fMRI data that have the same characteristics as the real data (number of volumes = 198, TR = 3.6 s, TE = 0.05 s), (ii) synthetic (smooth Gaussian) noise was added to the deterministic response to match the signal-to-noise ratio (about one) of our real fMRI data. This means that the proportion of variance explained by DCM is around one half, and (iii) the posterior parameter estimates (of one subject) were used as the “true” connectivity parameters to generate the synthetic timeseries (for more details see Fig. S1 of the Supplementary Material). These synthetic fMRI timeseries were then used to invert DCMs that included two driving regions and a fully-connected endogenous connectivity matrix—in the same way as for the empirical data (i.e., by varying the number of modes from 1 to n). This procedure was repeated ten times (i.e. ten different datasets of *n* = 20 timeseries).

## Results

**Fig. 3:**
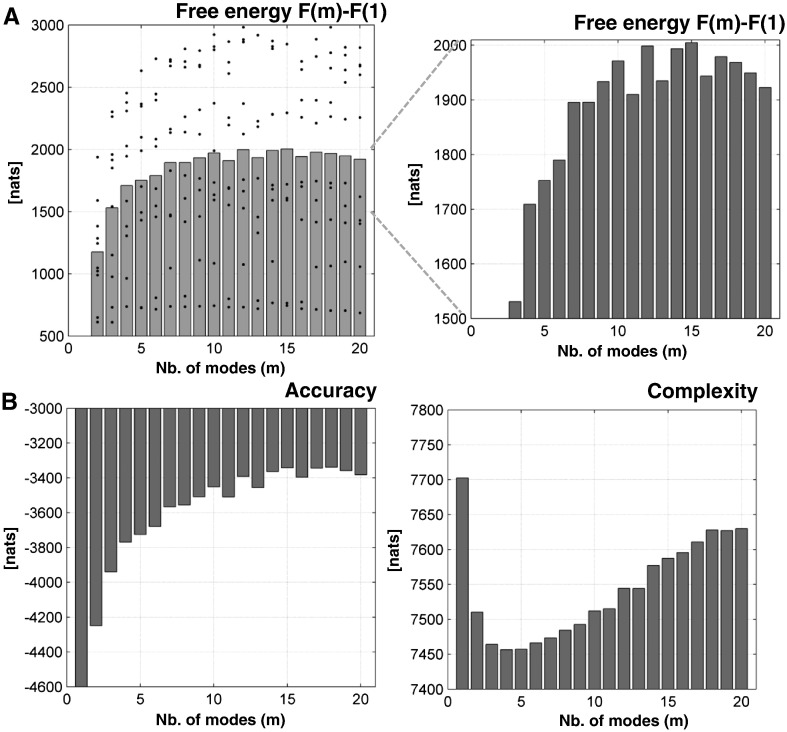
(A) plots the average free energy over our 10 subjects (that constitute a fixed-effect group model comparison) over the number of modes *m*—relative to the free energy at *m* = 1 (gray bar graph). The value of the free energy for each subject is shown as black dots. A zoom of the free energy (right bar graph) illustrates how the free energy (log evidence) initially increases with the number of modes and then decreases with larger numbers of modes. The winning model was at *m* = 15 with a difference in log evidence of 6, in relation to the next best model at *m* = 12. (B) illustrates the increase of the accuracy with the number of modes (left bar graph) but in the context of increasing model complexity (right bar graph). The complexity is just the difference between the free energy and accuracy.

Fig. 3 shows the free energy bound on log model evidence as a function of the number of modes averaged over all 10 subjects. Although the log evidence profile is not as smooth as one might like, there is a plateau at intermediate number of modes. This was the case for the (fixed-effect) group model comparison, with a peak at around *m* = 15. In other words, under the formal constraints of the model, the best explanation for these data is in terms of connections among 15 distributed patterns or modes. Note how the free energy falls systematically as the number of modes increases, after this peak. This particular pattern was observed in all but one subject (see subject-specific results in Fig. S2 of Supplementary Material). This reflects the fact that we are relaxing prior constraints on the extrinsic coupling by increasing the span of the prior covariance matrix. In effect, the model becomes over parameterised and its complexity starts to dominate (see Fig. 3). However, as we increase the number of modes, the model has more latitude to explain data and provide a more accurate prediction. In other words, we would expect the accuracy to increase monotonically with the number of modes. This is shown in Fig. 3 at the group level—and was seen in every subject (see Fig. S2 of the Supplementary Material).

**Fig. 4:**
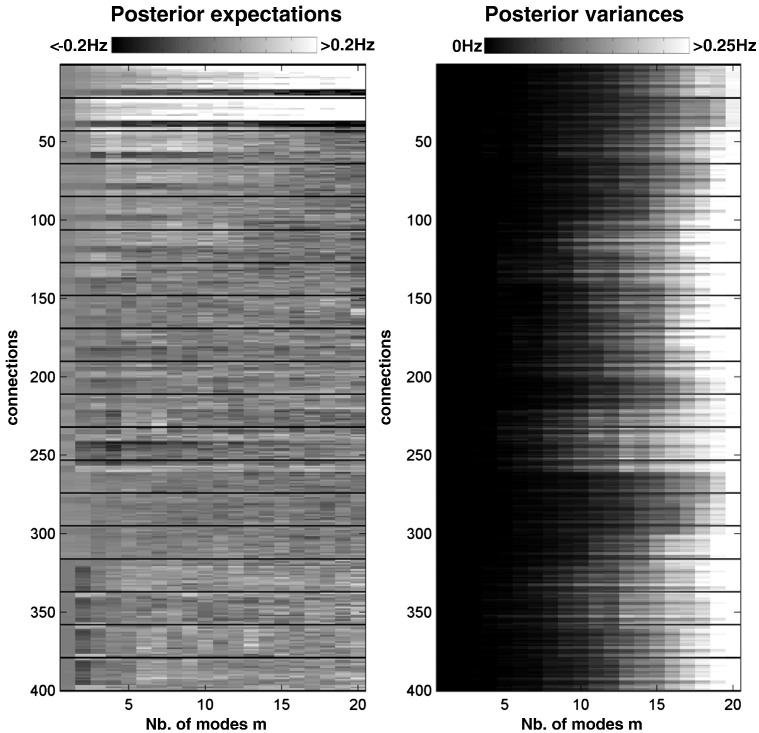
Posterior expectations (left) and variances (right) of endogenous coupling parameters over different number of modes. These were assessed at the group level by estimating the average posterior parameters and the pooled variances over 10 subjects using Bayesian parameter averaging. Each row of the connectivity map represents one connection among the 400 connections of our fully connected models (with the self-connections displayed as dark lines). The strength of some connections (i) increased monotonically with the number of modes (e.g. connections 2 to 40 with the driving regions), (ii) higher at intermediate number of modes (e.g. connections 50 to 60 or connections 240 to 250), and (iii) higher for *m* = 20 (e.g. connection 160). The posterior variances however showed a consistent pattern across connections, increasing with the number of modes (right graph).

As the number of modes increases – and the prior covariance constraints are relaxed – one would expect the posterior covariances to increase. Fig. 4 shows the posterior expectations and variances for all endogenous coupling parameters, as a function of the number of modes. The right panel shows that, as expected, posterior variance increases with the number of modes and loss of prior constraints. The behaviour of the posterior expectations in the left panel is interesting: some coupling parameters are high with a few (e.g., three to eight) modes, presumably because they have to explain most of the influence of modes on each other. Other sets of parameters seem to increase monotonically with the number of modes, as clearly evident for the connections with the driving regions (e.g. see details in Fig. 4). In situations in which differences in log evidence are not very strong (e.g. less than five (Kass and Raftery, 1995)), one could consider Bayesian averaging over models (Penny et al., 2010); such that the posterior expectations are weighted by the respective evidence for their model. However, in our case, the winning model had a sufficiently high log evidence to make this Bayesian parameter averaging unnecessary (as shown in Fig. 3).

**Fig. 5:**
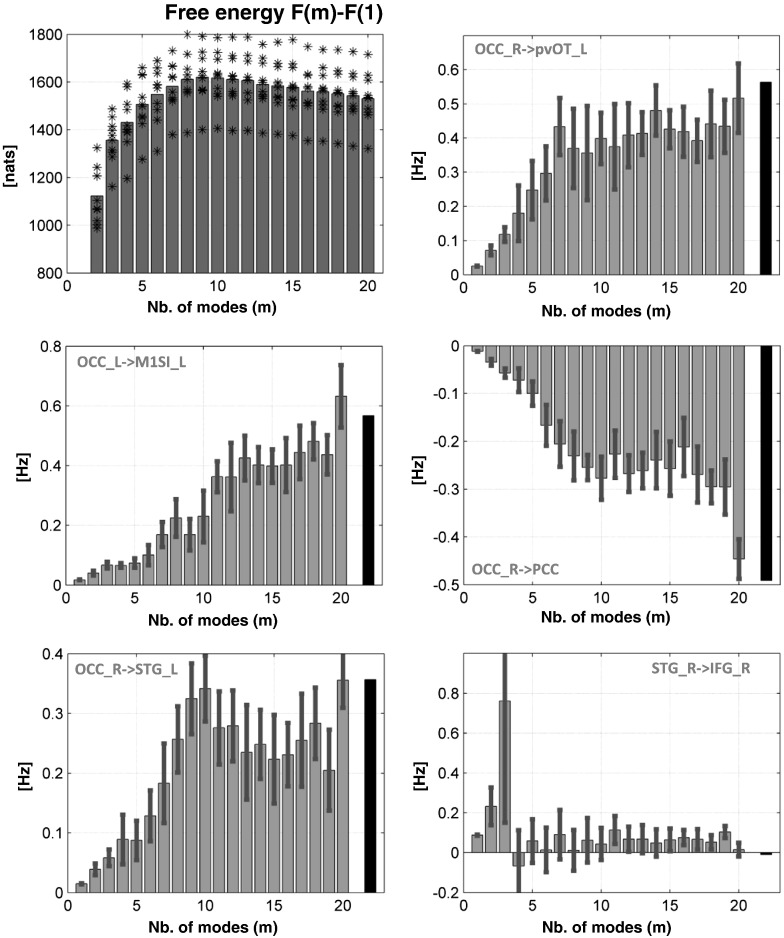
(Top-left) plots the average free energy of the 10 simulated datasets over the number of modes *m*—relative to the free energy at *m* = 1 (gray bar graph). The value of the free energy for each dataset is shown in black stars. The maximum free energy is clearly identified at intermediate *m* values. The remaining bar graphs (from top-right to bottom-right) illustrate the posterior expectations (in gray) after inverting the simulated DCMs (the bars in black represent the true coupling values used during the generation of the synthetic data). The error bars represent 95% confidence interval for the average over the 10 synthetic datasets.

This behaviour was confirmed in our modelling of simulated data. Specifically, across the ten different synthetic fMRI datasets, we found that the free energy was greatest at an intermediate number of modes (see Fig. 5). Fig. 5 shows the posterior means of exemplar coupling parameters. As in the real fMRI data, some parameters increased with the number of modes, while most remained close to the true value. A few parameters (e.g. bottom-right panel of Fig. 5) had inflated values with a small number of modes (e.g. *m* = 3), as seen with the real fMRI data (Fig. 4). We note that, for the model with unconstrained priors (*m* = 20), the posterior estimates of the coupling parameters were very close to their true values. Heuristically, differences between true and posterior estimates can be regarded being produced by a projection of the true values onto the parameter space spanned by the prior covariance matrix. Provided the true values lie in (or near) this space, the differences will not be large. The correspondence between the true parameters and the posterior estimates, for an intermediate number of modes, suggests that these modes produce a parameter subspace that (nearly) contains the true values. Fig. S3 of the supplementary material provides a detailed illustration of all connections, under different numbers of modes.

**Fig. 6:**
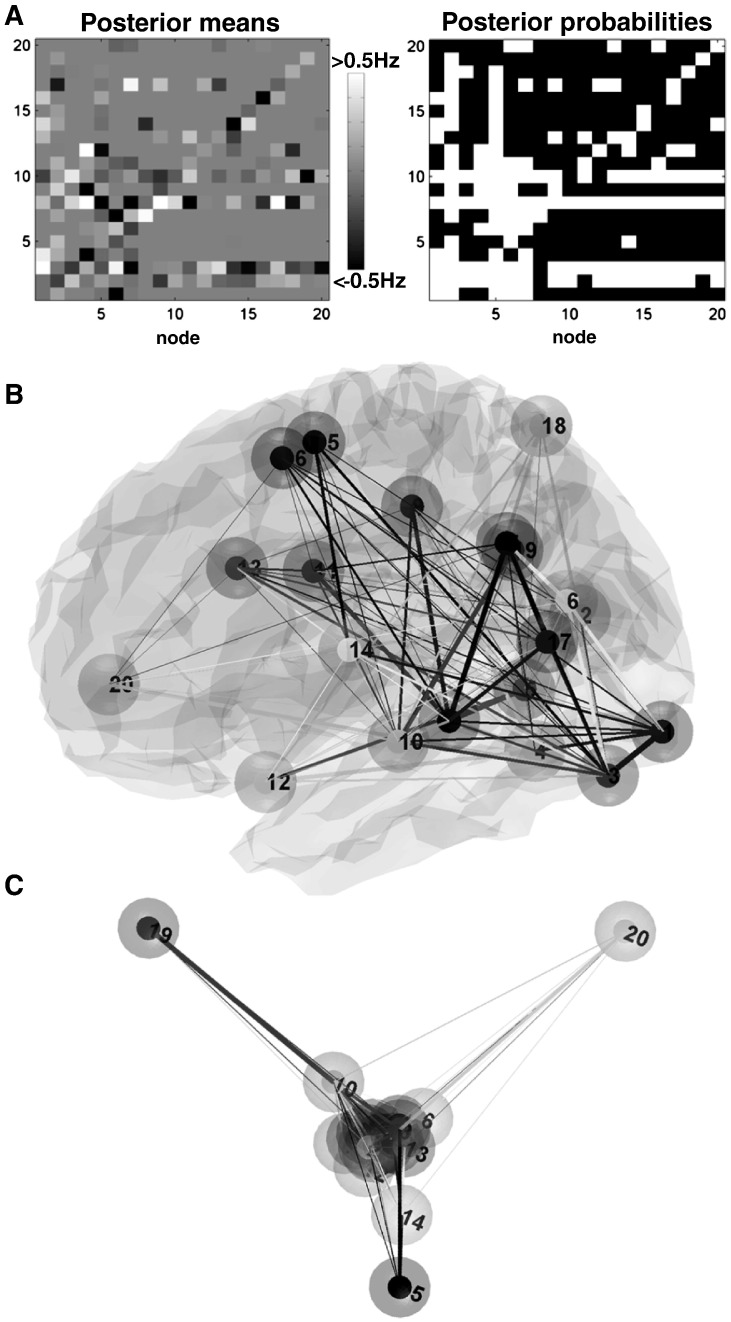
(A) The strength of the endogenous coupling parameters (displayed as a 20-by-20 connectivity matrix) of the reduced model after post-hoc model optimisation (left) and their posterior probabilities thresholded at *p* > 0.95 (posterior confidence—right). The coupling parameters of the reduced model ranged from − 0.7 Hz to 0.8 Hz. Both maps can be used to generate weighted or unweighted adjacency matrices respectively for graph theory analyses. (B) provides a description of the structure (or graph) of the reduced model in anatomical space. Here, we defined a weighted adjacency matrix that indicates the maximum between the absolute coupling parameters of a given connection and its reciprocal connection, excluding the self-connections (for a similar rationale see Friston et al., 2011). (C) provides a projection of the nodes into a functional space – using spectral embedding – where the distances reflect the strength of (bidirectional) coupling. The functional space was defined here using the first three principle components of the weighted adjacency matrix (c.f. Pages 1214–1215 of Friston et al., 2011). Regions are labelled from 1 to 20 in the same order as in Table 1.

The parameter estimates of the reduced model of the real fMRI data after post-hoc model optimisation are shown in Fig. 6. Starting from a fully connected model structure, post-hoc optimisation revealed a sparse model structure after identifying 247 anti-edges (Fig. 6). For instance, this revealed a striking difference between left cerebral versus right cerebellar nodes, with left temporal regions acting as target regions—receiving inputs from almost all nodes (e.g. left posterior ventral occipito-temporal cortex and posterior superior temporal gyrus), whereas the right cerebellum acted as a source region sending inputs to other nodes. The structure of the reduced model is also shown in a functional space, where the distance between nodes reflects the strength of bidirectional coupling (using spectral embedding as detailed in Friston et al., 2011). This revealed for instance a difference in the strength of bidirectional coupling between the core midline regions of the default mode network (as hubs (Andrews-Hanna et al., 2010)), with the other nodes of the reading system (see nodes 19 and 20 of Fig. 6C). We will not comment further on this connectivity graph; however, many readers will note that the underlying weighted adjacency matrix, encoding corrected connectivity, lends itself to several interesting analyses both in terms of functional anatomy and formal graph theoretic analyses.

## Discussion

In this paper, we addressed the Bayesian inversion of large graphical models using constrained priors that bound the number of effective free parameters. The implicit problem of over parameterisation was finessed by replacing priors on coupling among nodes with priors on coupling among modes—where modes correspond to the principal components or eigenvectors of the functional connectivity matrix. This reduces the dimensionality of the model inversion problem and incidentally speeded up the inversion scheme. Crucially, because the estimated coupling parameters are conditional on the model selected, it is important to ensure optimal posterior estimates under the best models that have the most evidence. Our findings showed models with a smaller number of modes – relative to number of nodes – had a greater model evidence, where the optimal number of modes appears to be around 15, for the data we analysed.

Although functional connectivity cannot identify directed interactions within a system (e.g. see recent discussion in Friston, 2011b), our results suggest that models with functional connectivity constraints provide better explanations for distributed responses than models without constraints. However, it is important to note that the functional connectivity does not bias the estimates of effective connectivity—functional connectivity was used to constrain the prior covariance of the model parameters, as opposed to their prior mean or expectation. Interestingly, this is exactly the same as the use of (undirected probabilistic) anatomical connectivity, when using diffusion weighted imaging or tractography data to inform dynamic causal models (Stephan et al., 2009). The use of such informative constraints ensures that each node still has its own intrinsic connectivity and haemodynamic parameters, while enabling DCM to provide unbiased and more efficient estimates of effective connectivity among nodes. The notion that functional connectivity may provide useful constraints can be motivated by the fact that strong statistical dependencies between measured timeseries are likely to be mediated by directed (effective) connections. More exactly, if two nodes (or sets of nodes) are statistically independent and show little functional connectivity, one can assume a priori that their effective connectivity is small. This prior assumption is embodied by the constraints on the prior covariance matrix. Our findings, with both real and synthetic fMRI data, confirmed that functional connectivity did indeed provide informative constraints (models with higher evidence) when inverting large DCMs.

We now turn to the impact of the number of modes on the posterior estimates of the coupling parameters. As illustrated with both real and synthetic fMRI data, using constrained priors (that bound the effective number of free parameters) changes the posterior expectations of the coupling parameters (Figs. 4 and 5). The results in Fig. 5 suggest the following. (i) The true values of coupling parameters lie within the 95% confidence interval with the full (*m* = 20) number of nodes, i.e., without prior covariance constraints. This confirms that unconstrained (in terms of prior coupling parameter covariance) inversion provides unbiased posterior estimates. (ii) However, for the intermediate number of modes (e.g. *m* = 8–12) where free energy is greatest, some true coupling values lie outside the 95% confidence interval. (iii) Moreover, for those coupling parameters, the true coupling values lie inside the 95% confidence interval only for the full number of modes. This would seem to indicate that maximising model evidence can lead to bias in the posterior estimates of some parameters. This is an inherent aspect of Bayesian model section, because changing the model is equivalent to changing the priors, and increasing prior constraints generally shrinks or biases posterior estimates towards their prior expectations (c.f., Stein shrinkage estimators in a classical setting or shrinkage priors in a Bayesian setting). More specifically, some connections (e.g. those connecting the two driving nodes) appeared to decrease in amplitude with prior constraints and the number of removed minor modes. This means that Bayesian estimators may underestimate the strength of some endogenous coupling and this should be considered in any quantitative interpretation. However, parameter shrinkage is generally not problematic in terms of inference about models. Indeed, one can see from Fig. 4 that the posterior variances also shrink with the number of removed minor modes, suggesting that significant connections (i.e. with significant posterior probabilities) are retained when using more informative priors.

**Fig. 7:**
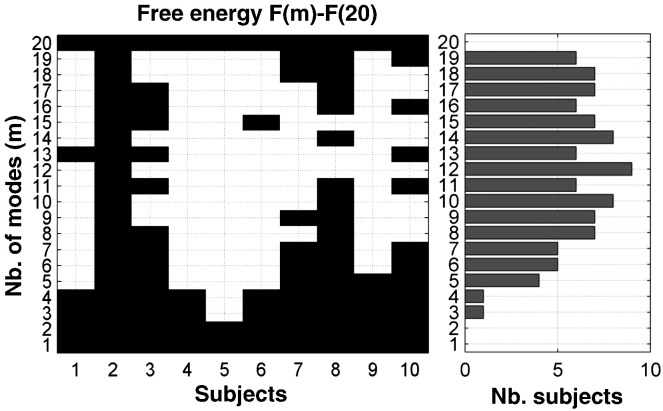
This figure illustrates the difference between the free energy at a given number of modes *m* and the free energy at *m* = 20 in each subject. Strong evidence (difference in log evidence of more than 5) is shown in white. The number of subjects that showed strong evidence at a given *m* compared to *m* = 20 is provided in the right bar graph, showing for instance that nine out of 10 subjects had DCMs with higher negative free energy at *m* = 12.

One practical question is whether one should optimise the number of modes for each new study. The procedures outlined in this paper describe how this can be done in a relatively straightforward fashion. However, we would not necessarily recommend inverting all possible DCMs for each new dataset. This is because of the computational load, particularly for studies with a large number of nodes or subjects. When looking at the range of *m* that ensured relatively high model evidences in each subject, it was clear that DCMs with a wide range of intermediate *m* values showed higher evidence (i.e. a difference in log evidence more than 5 (Kass and Raftery, 1995)) than the model with n nodes in almost all subjects (see Fig. 7). Put another way, there is a range of *m* values that ensure higher evidence for DCMs between modes than DCMs between nodes. Thus, for comparable group fMRI datasets, one might recommend between 8 and 16 modes for large models.

It is not unusual that fMRI activation maps contain multiple regions that one might want to explain in terms of distributed processing. The selection of candidate nodes for subsequent DCM analysis is generally motivated by one of two aims: network discovery through structural model selection (see discussion in Roebroeck et al., 2011) or testing hypotheses about specific connections. In the latter context, inferences on models with a small number of nodes are generally sufficient, given that conditional estimates of effective connectivity from a full graph are often consistent with estimates based on subgraphs (Friston, 2011a). In the setting of network discovery, our approach enables the inversion of large DCMs that can then be submitted to graph theory and related analyses. In principle, the analyses described in this paper can also be applied to resting state fMRI data with stochastic DCM (Daunizeau et al., 2009; Li et al., 2011). Although we have focused on deterministic DCM and standard activation paradigms, the procedures outlined in this paper can also be applied to stochastic DCM, using task free designs. Because stochastic DCM has to estimate both hidden states and parameters, it is computationally more intensive. This means the search for the optimal number of prior covariance (functional connectivity) modes may become prohibitive. However, this problem can be finessed using post-hoc optimisation (Friston and Penny, 2011). In other words, one would invert a full model (with no covariance constraints) and then evaluate the post-hoc model evidence (free energy) of reduced models, whose prior covariances have progressively fewer modes. Mode (model) selection using post-hoc optimisation appears to work extremely well for the deterministic DCM considered in this paper. The ensuing log evidence profiles are generally smoother (and possibly more reliable) than the estimates we have reported above. The estimates reported in this paper were obtained by brute force—inverting reduced models as opposed to reducing inverted models as described in Friston and Penny (2011). We will present this application of post-hoc optimisation – and related results pertaining to stochastic DCM of resting state timeseries – in a subsequent paper.

In conclusion, we hope that the ability to invert large DCMs, in an efficient way, will provide a new opportunity for analyses using graph theory—analyses that rest upon the statistics on a relatively large number of edges (Rubinov and Sporns, 2010). Furthermore, the ability to discover the structure of large models, using post-hoc model optimisation (Friston et al., 2011) may be valuable for studies of large and complex networks, in healthy and diseased populations. Our procedure can be combined with recent trends of using informed or tailored priors based on prior beliefs forwarded by models of anatomical connectivity—or on the basis of meta-analytic functional connectivity (see recent review in (Fox and Friston, 2012)). Future work needs to explore how our findings generalise to other DCM schemes, including stochastic DCM (Daunizeau et al., 2009; Li et al., 2011).

## Figures and Tables

**Table 1:** List of regions selected for DCM and their group coordinates in the MNI space. Regions of the default mode network are shown in *italic*. BA = Brodmann areas.

Region	Anatomical location	Coordinates (in mm)
OCC_L	Left middle occipital gyrus (BA 18)	− 20 − 92 − 6
OCC_R	Right middle occipital gyrus (BA 18)	44 − 82 − 8
pvOT_L	Left posterior ventral occipito-temporal cortex (BA 19/37)	− 38 − 78 − 10
Cereb_L	Left cerebellum (Lobule VI)	− 22 − 60 − 22
Cereb_R	Right cerebellum (Lobule VI)	22 − 58 − 24
SOG_L	Left superior occipital gyrus (BA 19)	− 26 − 70 32
SPL_R	Right superior parietal lobule (BA 7)	30 − 56 52
pSTG_L	Left posterior superior temporal gyrus (BA 22)	− 56 − 42 16
pSTG_R	Right posterior superior temporal gyrus (BA 22)	60 − 36 14
STG_L	Left superior temporal gyrus (BA 22)	− 62 − 20 12
STG_R	Right superior temporal gyrus (BA 22)	54− 16 4
IFG_L	Left inferior frontal gyrus (BA 44)	− 56 10− 4
IFG_R	Right inferior frontal gyrus (BA 44)	60 12 0
M1SI_L	Left primary motor and somatosensory cortex (BA 3/4)	− 48 − 14 38
M1SI_R	Right primary motor and somatosensory cortex (BA 3/4)	50 − 8 32
SMA	Supplementary motor area (BA 6)	0 − 2 58
*IPL_L*	*Left inferior parietal lobule (BA 39/40)*	− *50* −*72 30*
*IPL_R*	*Right inferior parietal lobule (BA 39/40)*	*48* − *68 38*
*PCC*	*Posterior cingulate cortex (BA 7)*	− *8* −*58 26*
*MFC*	*Medial prefrontal cortex and anterior cingulate (BA 10/24)*	− *2 40* −*10*
