# Supplementary material for: Network discovery with large DCMs
Source: Neuroimage. 2013 Mar;68:181–91. doi: 10.1016/j.neuroimage.2012.12.005 (PMC3566585; doi:10.1016/j.neuroimage.2012.12.005)

Figure S1: (Top) the true coupling parameters (in [Hz]) used during the generation of the synthetic fMRI data (the order of nodes is the same as in Table 1 of the main text). (Bottom) illustration of simulated fMRI timeseries of 20 nodes.

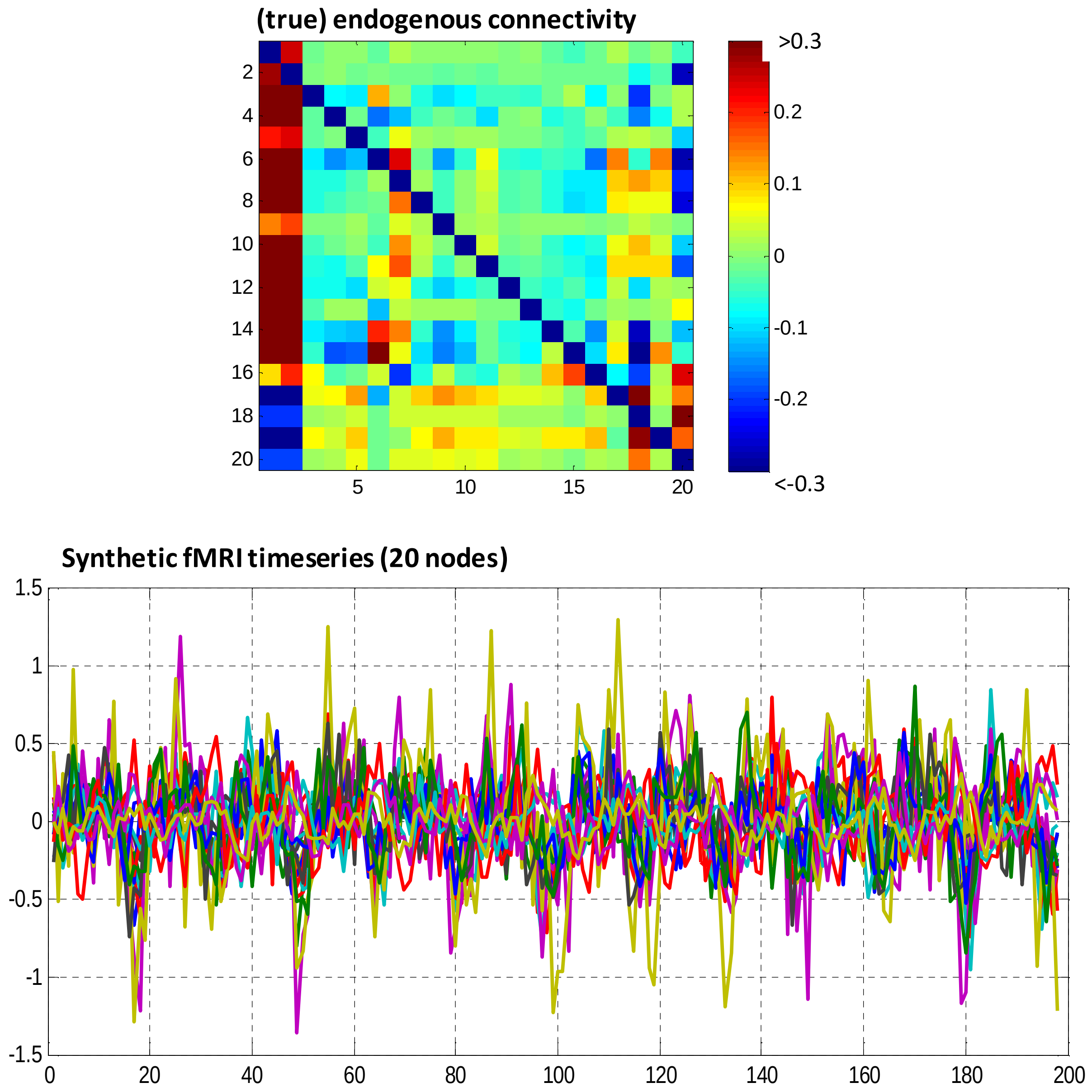

Figure S2: plots of the free energy (left), accuracy (middle) and complexity (right) of each subject after the inversion of the 20-node DCMs at variable number of modes.

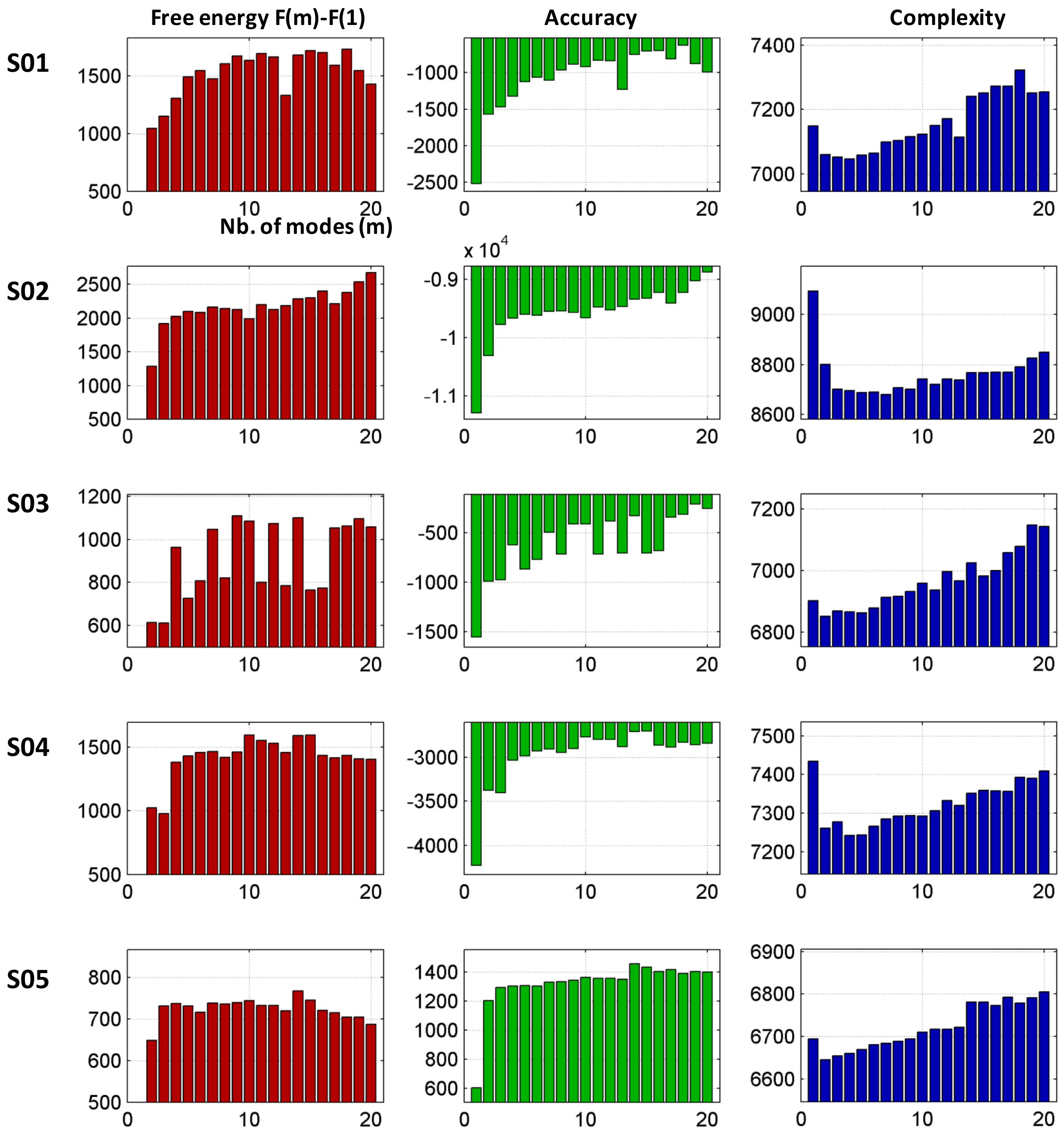

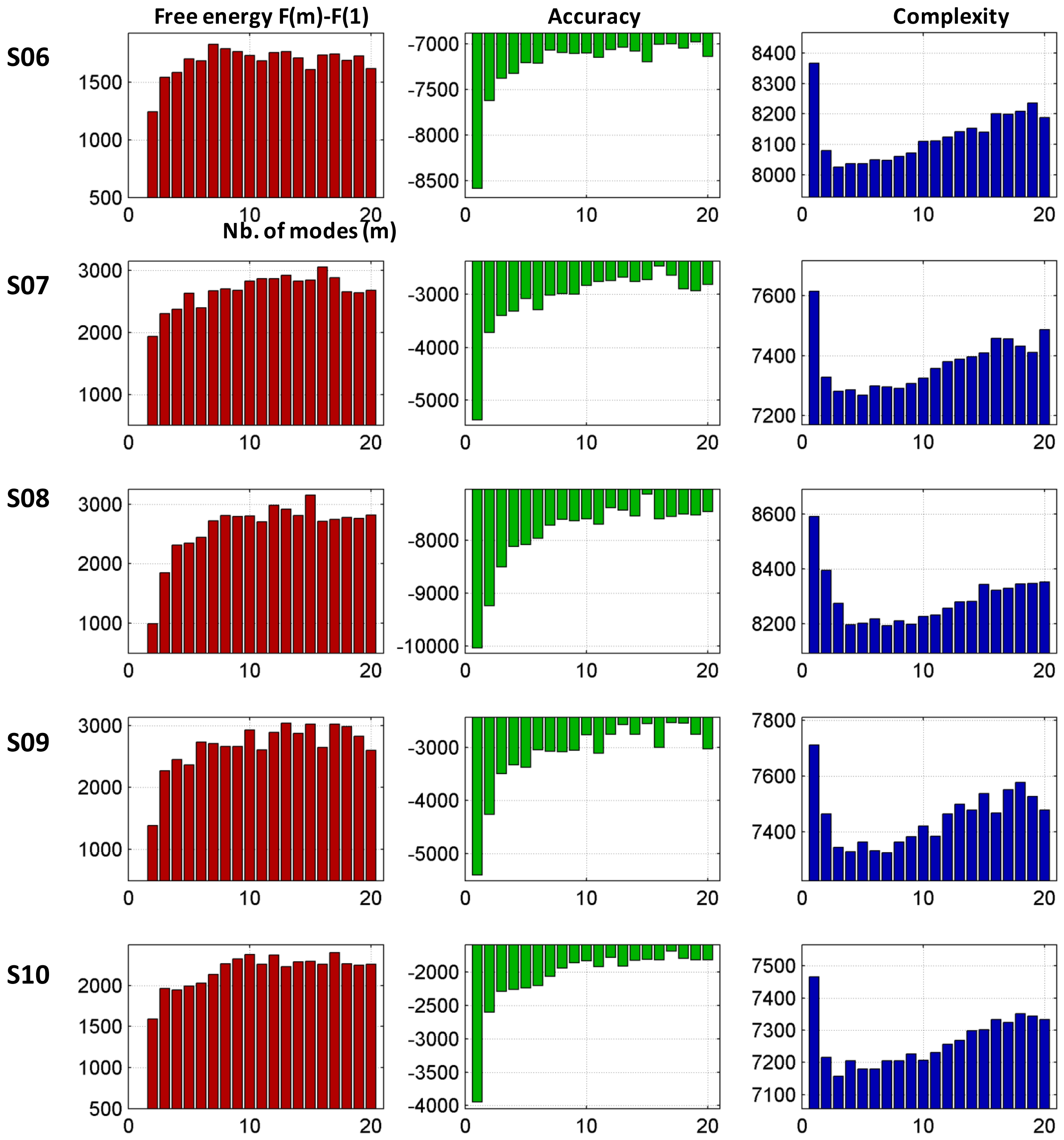

Free energy F(m)-F(1)
Accuracy
Complexity
S06
S07
S08
S09
S10
Nb. of modes (m)

Figure S3: scatter plots of the posterior estimates of the coupling parameters (y-axis) against the true parameters (x-axis) at each number of modes (m varied between 1 and n=20). There is a good correspondence at intermediate number of modes that showed higher free energy. The slope of the scatter plots seems to increase with the number of modes, which is in line with our findings on both real and simulated fMRI data where the strength of some connections increased with the number of modes (Figures 4 and 5 of the main manuscript).

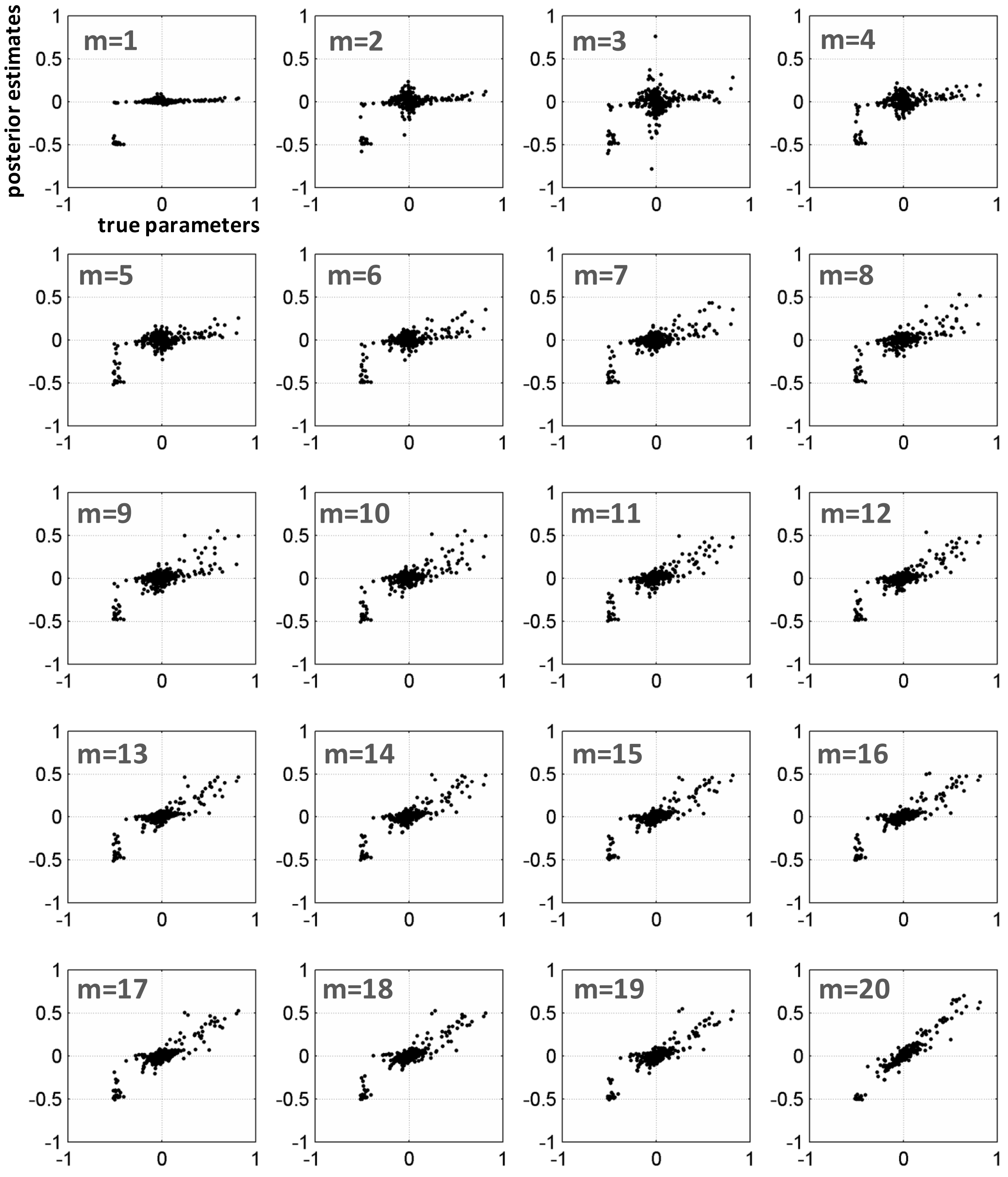

Supplement: Supplementary file 1 — Supplementary Figures [file mmc1.pdf]
